# On the Pathology of the Urine

**Published:** 1859-09

**Authors:** 


ART. II.—*A Treatise on the Pathology of the Urine; including a complete Guide to its Analysis.* By J. L. W. THUDICHUM, M.D., late Physician to the St. Pancras Royal Dispensary; Lecturer on Chemistry at the Grosvenor Place School of Medicine; with seven Plates. 8vo., pp. 430. London: John Churchill. 1848.

*Urinary Deposits; their Diagnosis, Pathology, and Therapeutical Indications.* By GOLDING BIRD, M.D., F.R.S. Edited by EDMUND LLOYD BIRKETT, M.D., Caius College, Cantab.; Fellow of the Royal College of Physicians; Physician to the City of London Hospital for Diseases of the Chest, etc. A new American from the fifth London Edition. 8vo., pp. 382. Philadelphia: Blanchard & Lea. 1859.

UNDER the above title Dr. Thudichum has added another contribution to urilogical science. The work is a large octavo, very handsomely illustrated. The manner in which it will be received will, no doubt, depend upon the wants of the reader. He who takes it up with the expectation of finding a treatise in full upon the pathology and treatment of the alterations of the urine, will be very much disappointed. Again, if the reader be one looking for originality and much new matter, we fear he also may lay it aside; while those who are desirous of a work on the urine, rather more scientific than practical, well compiled by one fully conversant with his subject, will very likely give it a place in their library. Some original matter, it is true, we find scattered throughout the book, but the impress is that of a compilation of the knowledge of the day.

The physical characters of the urine are first discussed. Its various tints are described, and attention attracted to the series of hues which may be produced by the different degrees of concentration or dilution of the normal coloring matters of this fluid; and as it is difficult to obtain, from simple description, an accurate idea of the different shades of colors assumed, it would be well for those who are studying this subject to imitate the different tints by the method of Vogel, which Dr. Thudichum has introduced into his work.

The following is the method given by Vogel for imitating these various colors. It also enables us, as remarked by Dr. Thudichum, to estimate the quantity of uræmatine with a tolerable degree of accuracy:—

I. *Group.* YELLOWISH URINES.

The color is yellow, (gamboge,) mixed with a greater or less amount of water. Starting with the perfectly colorless urine, this group has three gradations or *nuances*:—

1. Pale yellow, (gamboge, with much water.)
2. Light yellow, (gamboge, with less water.)
3. Yellow, (gamboge, with very little water.)

II. *Group.* REDDISH URINES.

With the yellow there is mixed a greater or less amount of red, (gamboge, with crimson lake.) These urines are generally termed "highly colored." There are again three varieties of the group:—

4. Reddish yellow. An admixture of some red to the prevailing yellow, (gamboge, with a little crimson lake.)
5. Yellowish red. The red color becomes more prominent in the yellow body of the fluid, (gamboge, with more crimson lake.)
6. Red. The red color is prevalent, but there is still a slight admixture of yellow, (crimson lake, with little gamboge.)

III. *Group.* BROWN (dark) URINES.

The red color passes through brown to almost black tints, (gamboge, crimson lake, and more or less Prussian blue.)

7. Brownish red. Red, with an admixture of a little brown.
8. Reddish brown. There is more brown color than in the last.
9. Brownish black. Almost black, but with a touch of the reddish brown.

In using this method, a unit is established, which, however, does not express an absolute weight. It is the amount of uræmatine or coloring contained in 1000 C. C. of the first variety, or pale-yellowish urine.

The objections which have been made to this method are met and answered, and the physiology and pathology of the normal coloring matter considered, which (normal coloring matter) is termed uræmatine.

The abnormal coloring matters peculiar to the urine, are urerythrine, the purpurine of Dr. Bird, and uroxanthine or Indican,—the uroxanthine being decomposable into uroglaucine, or indigo blue, and urrhodine, or indigo red. These are fully discussed, and much information is to be obtained by reading the chapters devoted to their consideration. We should think that the literature of this subject is fully exposed, and we regret the want of space to give some extracts from this part of the work. Our knowledge of these ingredients of the urine is, however, still obscure, but we hope that soon there will be more light thrown upon their history, both chemically and pathologically, for indications highly practical may be deduced from their observation in disease.

The author has treated this part of his subject in different portions of the work, but we have, for the sake of convenience, considered it *en masse.*

The author has presented the different tints of the urine, the coloring matters producing these tints, their pathological import, and their chemical tests, in a tabular arrangement. The odor of the urine is stated to be owing to the presence of a series of volatile acids, such as phenylic, (or carbolic,) taurylic, damaluric, and damolic acid.

*Chemical reaction of urine.*—The acid reaction of the urine is supposed to be owing to acid salts, such as the phosphates and sulphates of potassa and soda, the acid urates and hippurates, and accidentally to free organic acids, such as the oxalic, malic, and tartaric, and their acid salts. The observations of Bence Jones and Vogel are quoted, in regard to the variation of the acidity of the urine at different periods of the day. According to the former observer, the acidity of the urine being in an inverse proportion to the amount of acid secreted by the stomach during digestion, and according to Vogel, the highest amount of acid discharged with the urine per hour occurred during the night, the lowest in the forenoon, and the medium amount in the afternoon.

It is also mentioned that the neutral or alkaline condition is due not so much to the absolute absence of the acid, as to its neutralization by bases, and that these bases are the volatile fixed alkalies; the volatile alkali being derived from the arrangement of the elements of urea into carbonate of ammonia; the fixed alkalies being obtained often from the administration of the different salts formed by the union of the fixed alkalies with the organic acids which are transformed into alkaline carbonates in the system. Another cause of alkalinity from the fixed alkalies is, we would add, as Bird has mentioned, a hearty meal of *bread;* and we have had frequent opportunities to observe this in patients, who, after passing such urine for days and months together, always after breakfast have seen this condition vanish on diminishing or omitting their bread diet at their morning meal.

Attention is also attracted to the fact, that while healthy urine does not become ammoniacal for some hours after emission, yet we sometimes find it becomes so almost immediately after being passed; and this should be attended to, as it is of practical import.

While the ammoniacal condition of the urine is, as the author believes, invariably due to the decomposition of the *urea* in the urinary passages, yet he suggests the possibility, though it has not been proven, of this condition being owing to the direct passage of ammoniacal salts from the blood. The author also takes up the consideration of the various conditions of the bladder which may produce an alkaline state of this excretion.

A neutral and alkaline condition is often of frequent occurrence with the pale urine discharged in anæmic conditions; and attention is directed to the highly practical idea that this condition of urine indicates the employment of tonics, particularly iron.

Next in order are considered the changes produced on the urine by cooling, of the mode of collecting the urine, and of the quantity of urine passed in a given time. Here the author most justly criticises the habit of many observers heretofore, who have given the results of analyses of the urine without any regard to the amount passed in any given time; and we would add, that often we have been prevented giving a satisfactory answer to physicians who have sent specimens of urine to be examined, from the neglect of this observation.

The author presents us with a series of observations on the quantity of urine passed in 24 hours in two healthy adult individuals. In the one case for 76 days, with some interruption, and in the other for 55 days, also with some interruption. The average amount per day in the first was 1950 C. C., or 65 fluidounces; the minimum being 1049 C. C., the maximum 2920 C. C. The second series gives 1723 C. C. per day; the minimum 1040 C. C., the maximum 2655 C. C. He also compares with his own the results of the observations of Lecanu, Valentin, Lehmann, Bischoff, Vogel, and others.

The author here remarks: "The values obtained by my observations, extending over 133 days of two individuals, are throughout considerably larger than those of former observers. They may, therefore, be said to extend our knowledge of the average quantity of urine secreted by adults, and of the lowest and highest amount consistent with healthy functions." We would add that while the observations are numerous, they are nevertheless confined to but two individuals, and are consequently too limited to *satisfy* us.

Again, if our author means us to take the above results as expressing the average quantity of urine passed per day by healthy adults, we must beg to differ, at least so far as to take it as a standard for England, much less for this country. It is true, that for Germany and France, where light wines are much indulged in, this estimate might do.

There is a most marked difference between the above estimate and those of Prout and Dr. Routh. The former estimating the average quantity for 24 hours throughout the year to be 32 ounces; the latter, from observations in 18 cases, 35 ounces. Becquerel estimates 43 ounces for men, and 67 for women. Vogel, 54 ounces; Reuke, from a mean of 23 days in the months of September and October, gives about 41 ounces. Bence Jones remarks, that if 50 ounces of fluid be taken in 24 hours, the average quantity will be 44 ounces. We would add, from a considerable number of observations in this country, that it is not at all unusual to find persons passing below 32 ounces; and we have been led to consider a quart of urine to be about the normal average amount passed by the majority of healthy individuals, variations of course taking place from the habits of the individuals; the quantity depending upon the time of the year, the activity of the skin and other causes, and the amount and nature of the fluids *imbibed.*

The author, after noticing the variations in the quantity of urine in different portions of the day, speaks of the effect of disease in altering the quantity, and most justly remarks upon the high diagnostic and prognostic value of alterations of the amount of urine in disease.

The chapter on the solids and water, and the sp. gr. of the urine, is occupied in discussing the methods of estimating the solids and fluids of the urine. We will confine our attention to the points most interesting to the practical physician, among which is the value of the gravimeter in estimating the amount of solids. In the first place, the author remarks, there are two sources of error arising out of the use of these instruments, which must be corrected before determining the amount of solids after reading from the gravimeter. The first source of error is in the instrument itself, and consists in the division of the scale into equal parts. This, of course, goes upon the assumption that the strata of the fluids in which the gravimeter is immersed are all of equal density. But as the density increases from the surface, the graduations of the scale ought to be corrected upon the different densities of the respective volumes of urine.

The second source of error is the variable temperature of the urine. This last error, as the author remarks, is obviated by bringing the urine to a temperature of 60°, or to correct the reading of the gravimeter by a compensating calculation based upon the temperature found. For this purpose he refers to the table of Mr. Akland. "Ex. Suppose the urinometer to float in urine of 73° temperature at 21. On reference to the table opposite 73° will be found 1·20, which is to be added to the indication 21, making the true specific gravity =1022·2."

Formulæ for calculating the amount of solids in urine by the gravimeter are found in all books on the urine.

The author again states, "that in calculating the solids from the specific gravity, we are liable to commit an error, amounting to one-tenth or one-seventh of the solids actually contained in average healthy urine; and the error may be either above or below the amount actually contained. And in concentrated urines and the urine of disease the error may be still greater." Finally, the value of the gravimeter is summed up by the following: "What with having to employ two gravimeters to compensate and calculate for temperature and density from depth, and being after all subject to inaccurate reading, and the varying proportions of the solids in urine, errors which, in the aggregate, amount to a difference in reality of from one-tenth to one-fourth, I must say, that I think the trouble of the methods with the balance and air-pump nearly equaled, and yet the result of a hundred gravimetered analyses is scientifically and practically not worth one accurate analysis."

The above certainly seems to throw a damper upon those who rely upon the gravimeter in their examination of the urine; but we would remark, however such a statement as the above might compel the chemist, accurate up to his $\frac{1}{100}$ of a grain, to give up its use, it need not so affect the physician who examines urine for clinical purposes, and who must give a wide latitude in his calculations. Where the specific gravity is high, there is no doubt that if more than ordinary accuracy is needed, the gravimeter should not be relied upon.

The following table, drawn up by Dr. Bence Jones to illustrate the inaccuracy of the gravimeter, we think might be used to prove that the instrument is sufficiently accurate for bedside use, with the exception mentioned above. Column A represents the amount of solids found by actual analysis, B the amount indicated by the *gravimeter*:—

| | A. | B. |
|---|---|---|
| 1028·0 . . . . | found 67·03 grs. | by table 65·2 grs. |
| 1028·5 . . . | " 66·59 " | " 68·5 " |
| 1028·2 . . . . | " 64·77 " | " 65·0 " |
| 1034·3 . . . | " 84·65 " | " 79·0 " |
| 1024·7 . . . . | " 60·79 " | " 58·0 " |
| 1024·8 . . . | " 64·61 " | " 58·0 " |
| 1024·8 . . . . | " 56·67 " | " 58·0 " |

In his estimate of the amount of solids excreted daily by the kidneys, the author seems to agree more with the continental observers than with the English. He considers the daily excretion of the solids to range between 850 and 1020 grains; Bird's estimate being from 600 to 700 grains. We have not time or space to discuss this point, however interesting, but would simply state our opinion that the estimate seems to be too high. The remarks upon the fluctuation in the amount of solids in disease, both acute and chronic, are practical and interesting. One or two extracts will serve to indicate their nature:—

"In chronic disease, with a limited amount of solids, a rise in their amount is, as a general rule, a forerunner of considerable and mostly lasting improvement, and indicates that the chemistry of the body is actively employed in renewing the material of the organs and rectifying their functions. To this rule diabetes mellitus alone forms an exception."

Again:

"Under all circumstances, a constant and gradual decrease of the amount of solids is as unfavorable a symptom as the decrease of the total quantity of urine discharged, because it indicates the decay of life, and a probably fatal termination of the case."

We now have arrived at that portion of the work which treats individually of the various normal and abnormal substances which are found in the urine. Urea is said to be the principal product of the metamorphosis in the body of the nitrogenous food. Again we find the following:—

"But one great fact is undoubtedly established; namely, that as urea is the principal product of the metamorphosis in the body of nitrogenized food, the quantity of urea must stand in a direct relation to the quantity of food taken; or, if little or no food be taken, to the amount of nitrogenized component parts of the body disintegrated in the place of food. In this sense must be taken the expression that urea is the measure of dissimilation, if I may be allowed to use this term as the antithesis of assimilation. The amount of urea is the measure of the most important part of the change of matter in the system. The intensity of the change is expressed by the amount of the urea in the urine."

The process of "dissimilation" we suppose to be synonymous with that expressed by Prout as the secondary destructive assimilation.

We do not exactly understand in what relation the author stands, in his idea of the origin of urea, to the two opinions expressed by different authorities, such as Liebig, Bischoff, and others, on the one hand, and Lehmann, Bidder, and Schmidt, on the other; the former maintaining that this substance is obtained from the nitrogenous tissues alone, while the latter suppose it to be derived, first, from the albuminous food; second, from the albuminous tissue. Evidently, however, from the second quotation above, his views are more those of Lehmann, Bidder, and Schmidt. We have always expressed ourselves in the following manner concerning this vexed question: that the urea is obtained from the nitrogenous material of the blood, and that this material is obtained from two sources, the disintegrated tissues, (muscular,) and from the *nitrogenized food*. Its normal source, if we may so use the expression, being from the material of the metamorphosed tissues, while it is obtained from the food only when that food escapes assimilation, either from debility of that function itself, or from the food being taken over and above the requirements of the system. Practically, this view brings us to the following conclusion: that if an individual has eaten largely of nitrogenous food, more than the system requires, after which an excess of urea is passed, we can but infer that this excess was obtained from the unused material. If, on the other hand, we find an excess of urea excreted, where there has been no such indulgence, then we must infer that it is obtained from the tissues, and indicates a too rapid waste of the system.

The average amount of urea discharged in twenty-four hours is put down at from 30 to 40 grammes.

Urea is found in excess, says our author, in all acute febrile diseases, being sometimes double the amount discharged in health. This increase is a more important feature of the disease when the ingestion of nitrogenized matter falls to a minimum.

In diseases which are chronic, and accompanied by impaired nutrition, the amount of urea sinks below the average.

The diminution of the quantity of urea may, however, be due to the failure of the excretory, or activity of the kidneys alone, though, at the same time, an excess may be produced in the system. The excess is then retained in the blood.

For the diagnosis of urea in urine, and other animal fluids, Liebig's method, modified, is adopted, which, stated briefly, consists in precipitating the phosphoric and sulphuric acids by baryta, then filtering, evaporating, and extracting by alcohol.

If albumen be present, the fluid should not be boiled, in order to coagulate it, as has been directed; for in so doing a certain amount of urea is inclosed in its substance. To avoid erroneous conclusions, therefore, albuminous urine, after precipitation with the solution of baryta, should be extracted with absolute alcohol.

A full description of Liebig's method of making a quantitative analysis of urea is given, and also of the modifications necessary to be made on account of the presence of chloride of sodium, ammonia, alantoine, which latter substance, however, has not been discovered in human urine, although it is said to have been found in that of the dog. There are other nitrogenized substances which must be got rid of by a solution of sugar of lead before the test can be used.

Uric acid is another important ingredient of the urine, and we can but notice the more practical points in this chapter. For determining the amount of uric acid, nitric acid is advised to be used rather than hydrochloric, for two reasons: First, nitric acid is more apt to precipitate uric acid than hydrochloric; second, hydrochloric acid favors the acid fermentation and development of certain descriptions of confervoid vegetations, which act as yeast-cells on the urates, decomposing them very rapidly. This process is entirely prevented by nitric acid. If the urine contains albumen, it is best to use the acetic acid, or the common phosphoric acid. Again, if the urine is of low specific gravity, it should be concentrated. This latter direction is highly important to bear in mind, for without such precaution there will sometimes be no precipitate when there is quite an appreciable amount of uric acid present in the fluid. The volumetrical method of Dr. Scholz for estimating quantitatively uric acid is also given.

In regard to the differential diagnosis of the urates, the author remarks, that as they are generally mixed in a deposit, it becomes a difficult question to decide whether the fixed or volatile alkalies are more commonly combined with uric acid. Lehmann considers the mass of the lateritious deposits which take place in febrile urine to be urate of soda, while the late Golding Bird and Prout looked upon them as urate of ammonia. But however important such a decision might be to the chemist, to the physician it makes as yet no difference; for the inferences to be drawn, in a pathological and therapeutical point of view, are the same.

The medium amount of uric acid made by a healthy man, in twenty-four hours, is 0·5 grammes or 7·72 grs. An interesting and important fact is mentioned, which is that great fluctuations take place in the amount of uric acid passed in health. This exhibits to us the necessity of being careful in predicating disease, unless the variation, from what may be considered the normal amount, be very marked.

There is nothing new or especially worthy of note on the pathology of uric acid, with the exception of a table of Becquerel, showing the amount of uric acid in the urine in different diseases.

The author considers the uric acid to exist in the blood as an acid urate of soda or ammonia, and also to exist as such in the urine.

Dr. Bird's theory to explain the manner in which uric acid exists in the urine is considered entirely untenable; and the views of our author are supported with vigor.

A subject of much interest is touched upon, which is the deposition of urates in the urinary passages; and in another part of the work the remark is made that it is singular that the urates should be deposited in the urinary passages, when they are so very soluble in the urine at the temperature of the body. Two causes are assigned for the production of urates in the urinary passages: First, by the absorbing of the watery parts of the excretion while it is in the bladder, thus concentrating the fluid, "in consequence of which a certain amount of the urates passes out of solution, and forms a precipitate."

The second cause is, as our author states, an alkaline condition of the urine, thus causing a precipitate. This latter cause seems to us the principal, if not the only cause. And again, a question has often arisen in our minds in regard to the nature of the deposit which is then precipitated, whether it be the ordinary granular amorphous deposit, the common deposit of fevers and indigestion, supposed by Lehmann to consist for the most part of urate of soda, or whether it be that form which consists of larger spherules, bodies often very grotesque in form, with spicula protruding from them. We are disposed to believe it is the latter, and what leads us to this belief is, in the first place, that the latter form of deposit is less soluble than the former, occasionally very insoluble, and especially if the urine be ammoniacal. Dr. Thudichum mentions a case where the deposit existed in the urine when passed. In this case he had to draw the water off with a catheter. The form of the deposit he represents in a plate, and it is of the nature of the above-mentioned insoluble form.

There are other subjects of interest connected with uric acid treated of in the work before us, but we are necessitated to pass on to another subject. Much could be said, and of a highly practical nature, concerning the causes of the deposit of free uric acid, but space will not permit our doing anything else but allude to them. The doctor advances nothing new upon this point, but gives Scherer's views, who perhaps has paid more attention to this part of the subject than any one else.

We find not much new about creatine and creatinine, with the exception of some experiments of the author upon the quantitative examination of this material, upon which very few observations have been made. According to Dr. Thudichum, 0·305 grammes of creatine were discharged in twenty-four hours. This calculation is an average of twenty-six days in two individuals. The idea is also suggested, that the quantity of creatine, together with that of creatinine, might serve to indicate the intensity of any spasmodic or convulsive action. Another interesting fact mentioned, is the relation of creatine to the amount of fat; where there is much fat in the tissue there are found but traces of creatine.

In regard to hippuric acid, the author quotes the observations of several, which go to establish the fact that the existence of hippuric acid in the urine is much influenced by exercise and rest, and also by diet. Martini, although the above statements seem to be controverted by the observations of Roussin, also found that fatigued horses produce much hippuric acid, and little urea. Horses well fed and quiet produce little or no hippuric acid. The experiments, however, of Roussin are not satisfactory. No notice is taken of Hellei's experiments upon himself, who found that after feeding for a week upon rye-bread, the uric acid disappeared and was replaced by hippuric.

Leaving now the consideration of hippuric acid, we will but glance at the chapter on chlorine and the chlorides. For the diagnosis of chlorine in the urine, the nitrate of silver, and Liebig's method, by means of the nitrate of the protoxide of mercury, are given. Then follows quite a number of pages, which are full and suggestive, on the "daily average amount of chlorine discharged during health and in disease." We will but mention the results obtained from the observations on this substance :—

"1. *That the amount of chlorine discharged during twenty-four hours varies in different individuals*, undoubtedly depends mainly upon the fact that unequal amounts of chloride of sodium are ingested with the food by different persons.

"2. The amount of chlorine discharged by an individual varies on different days, according to and corresponding with the amount of chloride of sodium taken with the food.

"3. The largest amount of chlorine per hour is secreted a few hours after the largest meal of the day, the smallest amount is invariably secreted during the night, (sleep.)

"4. Mental and bodily activity increase the secretory activity for chlorine of the kidneys, at any time during the day or night. This is particularly striking in the morning."

The facts upon which the above assertions are founded, are also thought to give support to the conclusion of Barral, that chloride of sodium increases the elimination of the nitrogenized ingredients of the urine.

"5. That the discharge of chlorides is not only diminished in pneumonia, but also in all acute febrile diseases, and that during convalescence the amount rises sometimes above the normal average.

"6. That the presence of chlorine in sputa, at a time when it is absent from the urine, is not sufficient proof of a determination of the chloride toward the inflamed lung, a proposition which loses all probability from the partial or total disappearance from the urine of the chloride in all acute diseases.

"7. That the absence of chlorine in the urine in disease is influenced greatly by the absence of a small amount of food in the diet of patients.

"8. In dropsical and hydræmic conditions, an increase of the amount of chlorine is a favorable symptom."

From the results of the observations of Gruner, Clare, and Naubauer, the average amount of sulphuric acid discharged during twenty-four hours by healthy young men fluctuates between 1·5 and 2·5 grammes. The influence of diet upon the amount of sulphuric acid discharged is very marked, and its origin is not only from the sulphates in the ingesta, but also, no doubt, from the oxydation of the sulphur which enters into the constitution of albuminous substances.

In speaking of the pathological indication, Dr. Thudichum remarks, that if there can be no doubt about the origin of sulphuric acid, the determination of its quantity in the urine must be useful for determining the amount of disintegration of albuminous matter in the system in cases where the ingestion of sulphur in any form or combination is very low or altogether suspended.

The amount of phosphoric acid discharged in twenty-four hours is influenced, as in the case of sulphuric acid, very much by the nature and quantity of the food. At the same time, the excretion of phosphoric acid is not exclusively dependent upon the quantities introduced.

According to the observations of Winter, quoted by our author, 100 kilogrammes of urine discharge, on an average, 0·27 gr., and 100 centimetres, 0·1 gr. of phosphoric acid. An important fact which is mentioned is, that great fluctuations take place in the hourly amount of phosphoric acid excreted, the maximum being after the principal meal, which was taken at noon, the minimum during the morning.

The following, from Dr. Thudichum, exhibits the influences producing an increase or decrease in the excretion of phosphoric acid :—

"Different degrees or morbid changes of the secretory activity of the kidneys, actual disease of the kidneys, change in the mode of disintegration of matter in the organism, must be looked to as causes of the variation of the amount of phosphoric acid."

The doctor also remarks that a series of observations demonstrate that the same influences which govern the excretion of chlorine and sulphuric acid are active in the excretion of phosphoric acid.

The following table will also exhibit the influence of various diseases upon the excretion of phosphoric acid :—

| DISEASE. | Number of days observed. | Min. | Med. | Max. |
|---|---|---|---|---|
| Males. | | | | |
| Emphysema of lungs | ... | 0.6 | 1·3 | 2·3 |
| Chronic bronchorrhœa | 8 | 1·3 | 2·7 | 4·7 |
| Cancer of liver | 11 | 1·6 | 2·2 | 2·0 |
| Subacute rheumatism of joints | 18 | 1·7 | 2·4 | 3·1 |
| Hemiplegia consequent on apoplexy | 35 | 1·0 | 2·7 | 5·2 |
| Hydruria | 3 | 4·4 | 5·0 | 5·8 |
| Dropsy, under influence of diuretics; chlorides very much increased | 2 | ... | 1·8 | ... |
| Females. | | | | |
| Diabetes insipidus | 14 | 3·2 | 4·8 | 7·8 |
| Ascites | 15 | 1·7 | 3·0 | 4·7 |
| Chronic rheumatism | 7 | 2·7 | 3·3 | 4·2 |
| Spinal irritation | ... | 2·1 | 2·4 | 2·8 |
| Amenorrhœa | ... | 2·1 | 2·2 | 2·3 |
| Scrophulosis | ... | 2·6 | 2·5 | 5·2 |
| Tuberculosis of lungs | 10 | 1·5 | ... | 3·9 |
| Chronic erysipelas of face | 11 | 1·5 | ... | 3·6 |

The remaining part of the work treats of the following substances: the free acid of the urine; potassa and soda; lime and magnesia; iron; ammonia; carbonic acid; blood and its anatomical elements; hæmatine, or hæmato-globuline; chylous urine; casts of uriniferous tubules; albumen; pus; mucus; fat and oil; cancer-cells and tubercular matter; echinococcus hominis; spermatic filaments, or spermatozoa; bile and biliary matters in urine; leucine and tyrosine; xanthine; hypoxanthine; sarcine (carnine;) cystine; allantoine; grape sugar; acetone; inosite; urerythrine; uroxanthine, or Indican uroglaucine; urrhodine; phrenylic, or carbolic acid; damaluric acid; oxalic acid; lactic acid.

Then are considered what the author terms urophanic bodies, which are divided into—First, the urophanic organic acids; second, urophanic organic bases; third, urophanic inorganic bodies.

The acids are succinic, malic, tartaric, citric, tannic, toluylic, salicylic, camphoric, anisic, and cuminic.

The bases are quinine and quinidine, strychnine, and santonine.

The inorganic bodies are iodine, arsenic, and antimony, lead, mercury, and copper.

Some of the above-mentioned subjects would admit of a lengthened notice, if time and space permitted; but as we have already encroached upon our limits to a greater extent than was intended, necessity forbids but a passing glance at some of the more interesting and practical topics. Nor will we pretend to give anything but a brief analysis.

The chapter on the free acid of the urine is rather meagre, and from its interest in relation to the diseases of assimilation, deserves, we should think, a fuller discussion.

The following extract, from the chapter on potash and soda, is of interest:—

"There is only a very small quantity of potash present in the urine, by far the greater part of the acids being in combination with soda and the alkaline earths. The value which an accurate knowledge of the quantities of potash and soda would have, is evident from their relations to different parts of the animal economy; the salts of potash almost exclusively predominating in the muscles, and occurring in the blood only in very small quantities, while the salts, with soda as their base, prevail in the blood, and, it may be well assumed, form no part of the solids of the juices of the flesh."

*Lime and magnesia.*—Dr. Thudichum lays down the rule that deposits of the earthy phosphates can exist only in urine, exerting an alkaline reaction upon test paper; there, however, being one (questionable) case in which a deposit may occur in acid urine; namely, when urine contains little or no free acid there exists an acid reaction from the presence of chloride of ammonium, and in this case the deposit would consist of phosphate of magnesia, this salt being insoluble in chloride of ammonium, while it dissolves the phosphate of lime.

In regard to the latter salt, Dr. Thudichum says, that crystallized deposits of this substance have not been observed, but, at the same time, remarks that it is more than doubtful whether it does not enter into the chemical composition, or is an admixture of some crystalline sediments. Attention is also attracted to an important fact in the diagnosis of these deposits by the microscope, namely, their great transparency, which prevents their discovery unless great pains are taken in illumination.

The proportion in which the phosphate of lime in the urine stands to the phosphate of magnesia is said to be about two to one. The presence of a deposit of phosphates in the urine, or their appearance by heat, is mentioned as by no means indicative of an excess.

The effect also of meat as causing an acid condition of the urine, while vegetable diet makes it alkaline, is also mentioned, and is a highly practical consideration.

*Grape sugar.*—The qualitative tests of sugar in the urine, which Dr. Thudichum seems to place foremost for accuracy and delicacy, are bismuth and copper, with caustic potassa. No mention, however, is made of the fallacies incident to the copper test, while the reduction of the bismuth is said to be produced by no other constituent of the urine, either normal or pathological, which, being the case, gives an advantage to it over that of Trommer's, though perhaps Trommer's is the more delicate.

*Pathological indication.*—Dr. Thudichum seems to be very skeptical concerning the assertions of those observers who have testified to the existence of sugar in the urines of healthy persons; and also mentions that even concerning those cases of acute disease, in which the temporary presence of sugar is said to have been discovered, doubt has not been perfectly excluded.

The existence of inosite in the meat eaten by carnivorous animals is thought by the author to prevent the conclusion of Claude Bernard, that the sugar found in the liver is formed from albuminous substances.

*Oxalic acid.*—The discussion of this substance occupies quite a lengthy chapter. The part which deserves our more particular attention is that which treats of the physical character of oxalate of lime and its pathological import, and we only regret the necessity of noticing it so cursorily.

An important observation made by the author, and one which is directly opposed to that of other observers, among whom we find the late Dr. Bird, is that the octahedral crystals of oxalate of lime have the power of polarization. This idea of their non-polarizing power is said, by Dr. Thudichum, to have been based upon the assumption that they crystallized in the regular or cubic system. The following extract will give the results of Dr. Thudichum's experiments :—

"I have already remarked that pure oxalate of lime can readily be ascertained to possess the power of polarizing light. But the ordinary oxalate of lime, the octahedra, have a very faint polarizing power, which can only be brought out fully by reflecting a ray of the sun through the crystal lying between the two Nichol prisms, and excluding from the eye every other light but that coming from the crystal in the microscope. The explanation of the fact that the ordinary octahedra polarize very faintly seems to be that, as they are very flat, their principal axis is mostly standing almost perpendicular. Now, as a crystal polarizes the less, the more parallel with its principal axis are the rays of polarized light passing through it, it is probable that the octahedra polarize only little, because the polarized light passing through them is almost parallel to their principal axis. This may be one reason, though, no doubt, others coöperate in obscuring the polarization of light by these crystals when ordinarily illuminated.

"It is now clear that there is no optical reason for assuming octahedra and dumb-bells to be made up of different materials. And as there is not a shadow of chemical evidence for the supposition that the dumb-bells consist of oxalurate of lime, they must for the present continue to be ranged under the oxalate."

The views of Dr. Thudichum on the pathology of oxalate of lime are conveyed by the following extracts, which are printed in italics :—

"1. The presence of oxalic acid or any of its compounds in urine may indicate that oxalic acid or any of its compounds has been introduced into the stomach, as an ingredient of food, as a medicine, or as a poison.

"2. The presence in the urine of any considerable quantity of oxalic acid, in any form, for a long period of time, during which the ingestion of oxalic acid, in any form, into the stomach was excluded, indicates the existence of a disorder, the nature of which is, at present, entirely unknown.

"3. Oxalate of lime is frequently found, in not very small amounts, in the urine of patients recovering from severe diseases.

"4. The presence in urine of such small microscopical traces of oxalate of lime that their quantity cannot be estimated by quantitative analysis, is of no practical or pathological importance.

"5. The presence of oxalate of lime in the urine may indicate disease of the kidney, with which it is not rarely associated."

The treatise of the late Golding Bird, on urinary deposits, has, for a long time, been known to the medical profession, and stood foremost among the works on the subject; but, from the rapid advance of the science of urology, it has gradually become, in some respects, a book of the past; but even now we are not aware of any work, of the same practical nature, which can yet take its place; and we were pleased to hear that one of our pet books was not to be pushed to the farthest-off shelf of our library, but would, as formerly, have its place on our table, among our daily guides, by being brought up to the day,—this day of rapid advancement, in certain directions. The old-fashioned, tedious, and unsatisfactory method of analysis, we are all becoming wearied of; and additions to Dr. Bird's work, giving a full description of the volumetrical processes, would still perpetuate this excellent treatise.

Dr. Birkett, we hoped, had thus improved the work of his late friend; but we are sorry to say that, while he has added not a little new matter, this most important and practical information he has either but slightly hinted at or entirely neglected to give us.

The physician in daily practice is, above all others, the one who has used this treatise of Dr. Bird, and who most needs a prompt and easy method of analysis; and at present he can only find it in the use of the volumetrical process. We would also suggest, though perhaps somewhat out of place, that some of our intelligent druggists or chemists should prepare these test-solutions, so that physicians might obtain them. The learning to use them would be but a moment's work. Physicians would then be tempted to make use of the means presented to them, and disease would be studied with that accuracy which the day demands.

We have digressed somewhat, in the above remarks, from our path; our intention was merely to give a brief notice of the above treatise, more especially as the English edition of the same has been so ably reviewed, and its contents so fully exposed, in the pages of this journal, some time since.

Among the additions by the editor which attracted our attention more especially, was the account of the analysis made by Hassall, confirming those of the German chemists, who have determined that the deposit which is so common in fevers and indigestion, is not urate of ammonia, but consists, for the most part, of uric acid, combined with lime, potassa, and soda, with small quantities of ammonia.

The remarks on the physiology and pathology of sugar are entirely new; the late observations of Dr. Pavy are here presented.

The cuts, exhibiting the confervoid growths which occur in the urine, would, we think, have been much more beautiful and truthful if they had been copied from those accompanying Hassall's excellent paper in the Medico-Chirurgical Transactions.

At page 317, Dr. Birkett has added a plate, which is said to exhibit the fructification of the "penicillium glaucum," but we must confess our inability to see any such exhibition in said plate. We cannot close these brief remarks without noticing the fact, that the American publishers have given us a very neat and beautiful edition of this work.

